# Bardet–Biedl syndrome in a Syrian adolescent: a rare case report

**DOI:** 10.1097/MS9.0000000000003077

**Published:** 2025-04-22

**Authors:** Ammer Alabed, Shaghaf Alhallak, Tala Dakkak, Ahmad Jamal Alhaj Ali, Noel Beiruty, Kamal Alwannous

**Affiliations:** a Faculty of Medicine, Damascus university, Damascus, Syria; b Faculty of Medicine, Albaath University, Homs, Syria; c Faculty of Medicine, Hama University, Hama, Syria; d Faculty of Medicine, University of Aleppo, Aleppo, Syria

**Keywords:** Bardet–Biedl syndrome genetics, central obesity, mental retardation, postaxial polydactyly

## Abstract

**Introduction:**

Bardet–Biedl Syndrome (BBS) is a rare genetic disorder that affects multiple organs and presents with a variety of characteristics. It was found that a dysfunction in the cilia causes it, in addition to mutations in the genes involved in the composition of these cilia. We present a unique case of BBS in a 13-year-old male from Syria, characterized by a significant family history of scleroderma and a distinct clinical presentation.

**Case presentation:**

The patient presented with intellectual disabilities, post-axial polydactyly, and a history of delayed developmental milestones. The patient’s BMI was within the normal range. Laboratory investigations revealed increased TSH and positive calcium oxalate, without evidence of central obesity or blindness. The diagnosis of BBS was made with a moderate level of confidence. The patient was undergoing treatment with thyroxine, showing improvement in thyroid function and communication skills.

**Clinical discussion:**

This case highlights the manifestations of BBS and the importance of recognizing atypical presentations. Diagnostic criteria traditionally emphasize a combination of primary and secondary features. We discuss the challenges of diagnosing BBS without genetic testing and the implications for treatment in resource-limited settings. Additionally, we explore therapeutics in managing the patient’s condition.

**Conclusion:**

This case represents a unique presentation of BBS, expanding the understanding of its clinical variability in different populations. The findings underscore the necessity for individualized diagnostic approaches and comprehensive management strategies in patients with limited access to genetic testing. By documenting this case, we contribute to the growing body of literature on BBS, particularly in the Syrian context, highlighting the need for increased awareness and research into this challenging disorder.

## Background

Bardet–Biedl syndrome (BBS) is a rare genetic disorder that affects multiple organ systems^[^1^]^ The disorder presents with various characteristics and is caused by cilia dysfunction. It follows an autosomal recessive inheritance pattern.^[^1,2^]^ BBS was initially identified by Laurence and Moon in 1866, with further cases documented by Bardet and Biedl from 1920 to 1922^[^3^]^ with a prevalence rate of approximately 1 in 160 000 live births in Europe^[^4^]^. The primary manifestations include central obesity, post-axial polydactyly, retinal dystrophy, hypogonadism, learning difficulties, and renal malformations^[^1^]^. Secondary manifestations may consist of diabetes, polydactyly, syndactyly, strabismus, cardiac issues, speech difficulties, and ataxia^[^2^]^. The diagnosis of BBS requires the presence of four primary features or three primary features and two secondary features^[^1^]^, a new paper suggests that modified criteria for diagnosis can be at a moderate level of confidence if it includes at least two primary criteria^[^5^]^. In this paper, we describe an adolescent male with a unique presentation of BBS. To our knowledge, this is the first case in Syria described in the literature. This case presents unique symptoms alongside various diagnostic methods that do not rely on genetic testing, thereby enriching the existing medical literature with important observations.

**Highlights**

- Bardet–Biedl syndrome (BBS) is an uncommon genetic disorder that affects multiple organs.
- The first reported case of BBS in Syria.
- A case of BBS with unique diagnosis not dependent on genetics test and presents with a variety of characteristics.
- The dramatic improvement based on multidisciplinary collaboration in addition to treating the patient with thyroxine

## Case presentation

**Figure 1.**
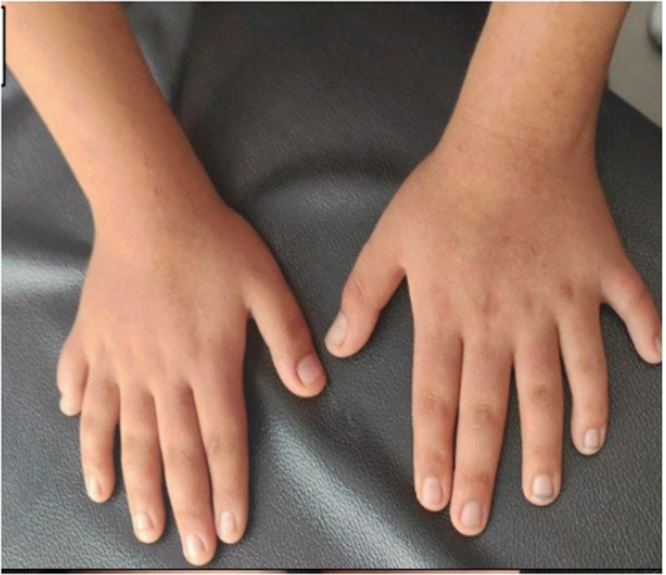
Right and left upper limb polydactyly.

**Figure 2.**
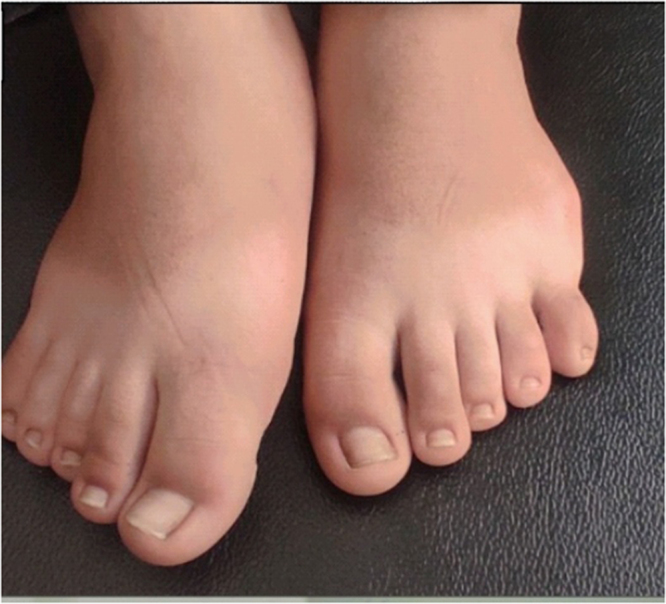
Surgical scar of phalangectomy of the lower limbs` sixth finger.

A 13-year-old male, from Homs, Syria was referred to an endocrinologist due to concern about his poor interactions with his surroundings since childhood. Additionally, the mother reported challenges in the child’s communication with friends, poor pronunciation, and subpar academic performance. Vital signs were within normal limits. He weighed 43 kg, with a body mass index (BMI) of 21.94 kg/m^2^, which falls within the normal range according to WHO classification. The mother noted that he had delayed developmental milestones compared to his peers. He experienced delays in both spiritual and motor development. The mother had full-term normal vaginal delivery by consanguineous parentage, Following his birth, he faced several complications, including cyanosis, cleft palate, post-axial polydactyly (Figs. 1 and 2), congenital hypoglycemia, hypothyroidism, uranoshisis, and cryptorchidism, with normal testicular size which was surgically corrected. The family history was significant for atrial septal defect, ventricular septal defect, and scleroderma; notably, his older brother died at 8 months due to scleroderma.

## Investigation

**Figure 3.**
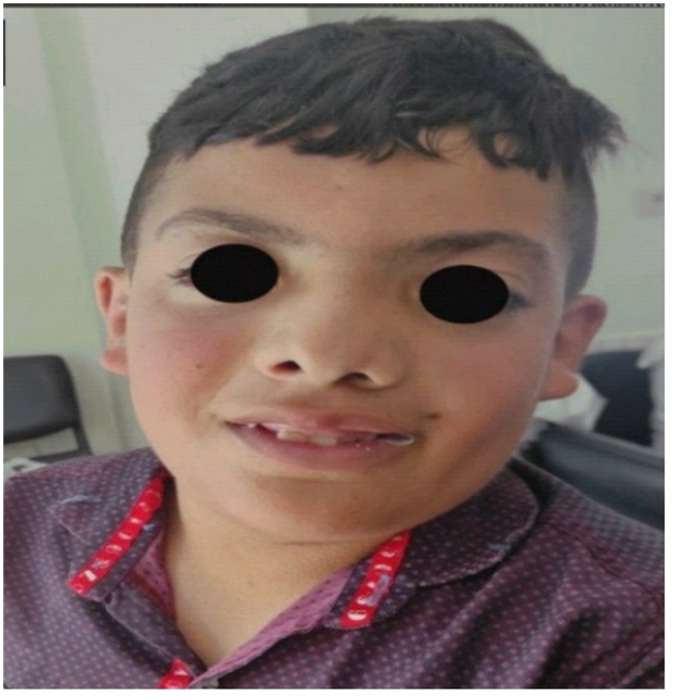
Gingival hyperplasia and a unique appearance.

Clinical examination revealed intellectual disabilities, gingival hyperplasia, and distinctive features resulting from cleft palate repair (Fig. 3). Additionally, he exhibited speech difficulties attributable to the surgery. Electrophysiological and genetic tests were not performed due to unavailability of resources. We performed laboratory tests, that included complete blood cell count, hemoglobin level, fasting blood sugar (FBS), phosphorus, and cylinder test, and all were within normal limits. However, the patient’s laboratory results indicated an elevated TSH level of 7.7, and calcium oxalate was found to be positive in urine. Moreover, he exhibited no signs of central obesity or a history of blindness.

## Outcome and follow-up

The thyroxine dosage has recently been increased to 100 mg, with subsequent findings indicating improvement in the patient’s condition and a decrease in TSH levels. Weekly follow-ups were conducted to further enhance his lifestyle.

## Discussion

BBS is characterized by a combination of symptoms including retinal dystrophy, renal abnormalities, intellectual disability, obesity, and polydactyly^[^1^]^. There were no indications of central obesity or a history of blindness, which sets this case apart from others documented in the literature. In contrast, One significant report examined a case that documented the occurrence of pigmentary retinopathy^[^3^]^. Another particularly noteworthy report focused on four diagnosed BBS patients, all of whom presented with renal abnormalities and varying stages of kidney disease^[^6^]^. In the absence of central obesity in our case study, it is important to highlight that another case reported in India discussed both intellectual disability and the presence of central obesity.^[^7^]^. This divergence underscores the importance of comprehensive evaluations in similar patients, as the absence of certain features, such as central obesity and blindness in our case, may suggest different underlying mechanisms or genetic factors at play. The causative mutations for BBS have been mapped to about 26 genes, however, mutations in BBS1 to BBS 18 in about 70–80% of cases worldwide, mutations in BBS1 and BBS 10 are detected in 21–30% of patients, a proportion that rises to 40–50% in Northern European patients. Mutations are involved in the function of the primary cilia in various cells and tissues of the body.^[^2^]^ Symptoms of BBS can vary widely among individuals, but the most common features include retinal dystrophy leading to vision loss, blindness, renal dysfunction leading to kidney failure, obesity, polydactyly (extra fingers or toes), intellectual disability, and hypogonadism^[^8^]^.

Diagnosis of BBS is typically based on the presence of multiple clinical features and genetic testing which involves analyzing the individual’s DNA for mutations^[^8^]^. However, in our case, genetic testing is not available. However, the slow development of clinical features makes it harder to diagnose^[^8^]^. The reliance on clinical diagnosis is often considered to hold greater significance than genetic diagnosis^[^9^]^. And in our case, genetic testing is not available in Syria. Now, however, relying on clinical diagnosis has greater value than genetic diagnosis^[^9^]^.

Sequencing the genes responsible for BBS using next generation sequencing (NGS) verifies the diagnosis in only 80% of cases^[^10^]^. A physical examination is where a healthcare professional looks for physical features commonly associated with BBS, such as obesity, vision problems, and extra fingers or toes^[^7^]^. Evidence supporting the diagnostic criteria proposes that a moderate level of confidence in diagnosis can be achieved when at least two primary criteria are met^[^5^]^. BBS is a multi-organ system disorder, and management, and treatment require a multidisciplinary approach. This may involve a team of healthcare professionals including ophthalmologists, nephrologists, endocrinologists, geneticists, and psychologists.^[^11^]^, While there is no cure for this condition, identifying it early and providing symptomatic, supportive, and rehabilitative treatments can help reduce its impact^[^12^]^. Orlistat and bupropion can help reduce body weight^[^10^]^. Some patients may benefit from Pharmacological interventions, such as the use of setmelanotide for obesity, which have shown promising results in improving quality of life and reducing body weight^[^13^]^.

Androgen replacement therapy is suggested based on the guidelines for both children and adults with hypogonadism^[^10^]^.

In our case, like many developing countries, may face challenges in terms of availability and affordability of these resources. Research should not only be focused on understanding the genetic causes, and mechanisms of BBS but also on developing an effective diagnosis method for this disorder.

Medical care involving ophthalmologists, nephrologists, endocrinologists, and baseline investigations that need to be done include blood sugar level, renal function tests, abdominal ultrasound, and intravenous pyelogram (IVP) are crucial for the diagnosis and the follow-up.

Support systems for individuals with BBS play a crucial role in providing comprehensive care, including educational resources, and psychological support. Which work to make life better for people with BBS and their families.

## Conclusion:

BBS affects individuals from diverse backgrounds and can place a worry on families. Support individuals with BBS helps provide comprehensive care, including medical support, educational resources, and psychological support. Which work to make life better for people with BBS and their families.

## Data Availability

The datasets used in this report are available from the corresponding author on reasonable request.

## Research registration unique identifying number (UIN)

ID: NCT06615011 (Unique Protocol ID: BBS syndrome).
